# Migrated embolization coil as a nidus for recurrent renal stones: A case report

**DOI:** 10.1016/j.eucr.2026.103374

**Published:** 2026-02-13

**Authors:** Thanisorn Pattanasuwon, Maria Camila Vargas, Manoj Monga

**Affiliations:** a Department of Urology, University of California San Diego, 9500 Gilman Drive, La Jolla, CA, 92093, United States of America; b Department of Urology, Phramongkutklao Hospital, 315 Ratchawithi Road, Thung Phaya Thai, Ratchathewi, Bangkok, 10400, Thailand

**Keywords:** Embolization coil migration, Renal stone, Ureteroscopy

## Abstract

Migration of embolization coils into the urinary collecting system is an exceedingly rare complication that may lead to recurrent urolithiasis. We report a case of recurrent renal stone formation caused by intraluminal migration of an embolization coil following selective renal arterial embolization performed for post–percutaneous nephrolithotomy (PCNL) hemorrhage. During ureteroscopic stone management, the coil was identified within the renal collecting system, serving as a nidus for stone formation, and was partially removed endoscopically. Recognition of this rare complication is essential for accurate diagnosis and appropriate management in patients presenting with recurrent renal stones after renal arterial embolization.

## 1 Introduction

Selective renal arterial embolization is an effective and widely accepted treatment for post-PCNL hemorrhagic complications, including pseudoaneurysm and arteriovenous fistula. Although generally safe, delayed complications may occur. Migration or erosion of embolization coils into the urinary collecting system is extremely uncommon but has been reported to result in foreign body encrustation and recurrent stone formation 1, 2, 3, 4. We present a rare case of recurrent renal calculi caused by a migrated embolization coil discovered during ureteroscopic lithotripsy.

## 2 Case presentation

**Fig. 1:**
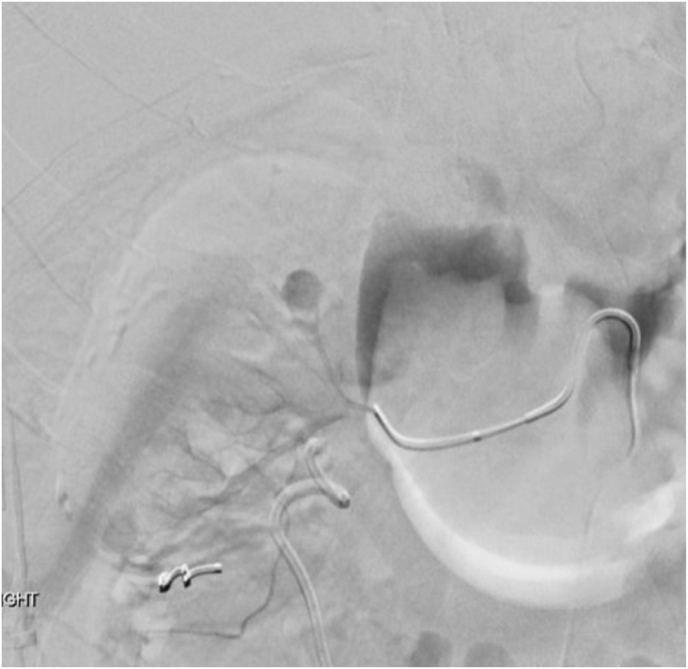
Angiography demonstrated a renal pseudoaneurysm.

**Fig. 2:**
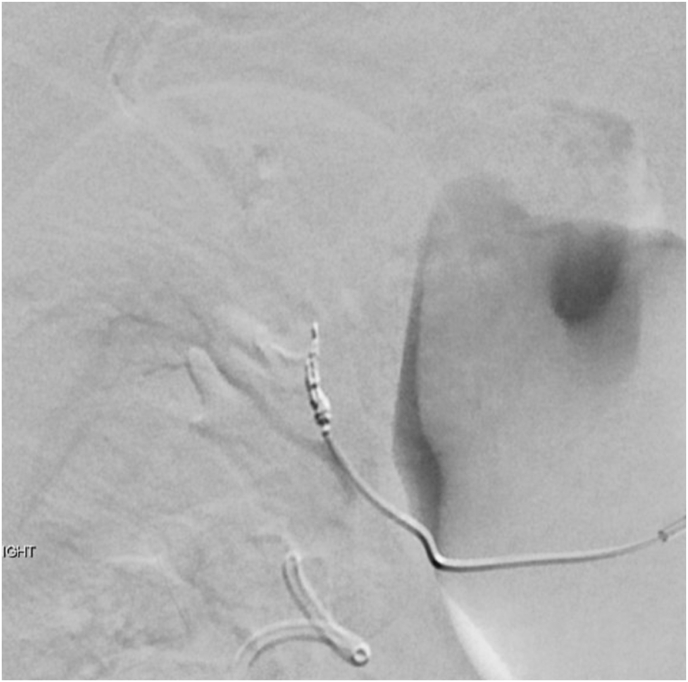
Selective renal arterial coil embolization was performed.

**Fig. 3:**
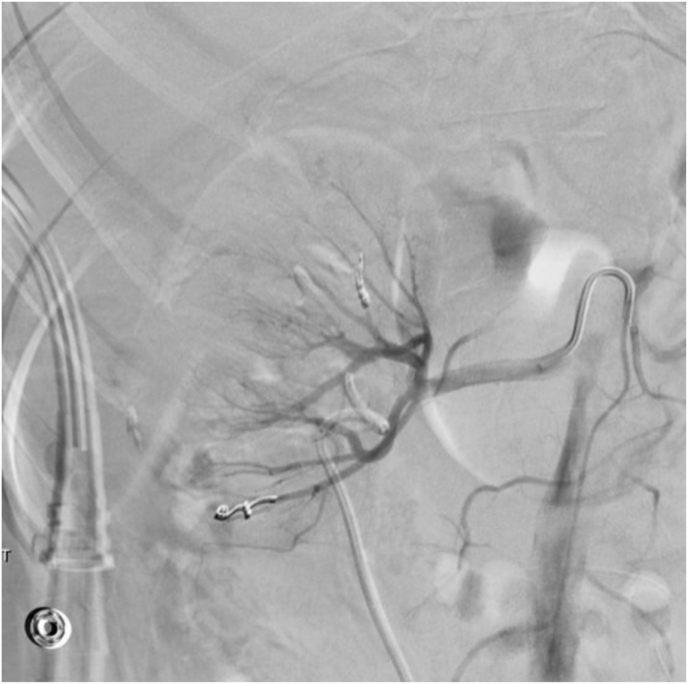
Post-embolization angiography.

A 42-year-old male presented with recurrent right-sided flank pain and renal stone disease. He had undergone right-sided percutaneous nephrolithotomy (PCNL) three years earlier for cystine renal calculi, which was complicated by significant postoperative bleeding. Subsequent angiography demonstrated a renal pseudoaneurysm, and selective renal arterial coil embolization was successfully performed (Fig. 1, Fig. 2, Fig. 3).

Review of procedural records revealed that embolization was performed using a Medtronic Concerto™ 3D detachable embolization coil (2 mm × 4 cm). This coil incorporates synthetic fibers to enhance thrombogenicity; based on manufacturer specifications and operative documentation, the coil used in this patient was most consistent with a nylon fiber–containing configuration.

**Fig. 4:**
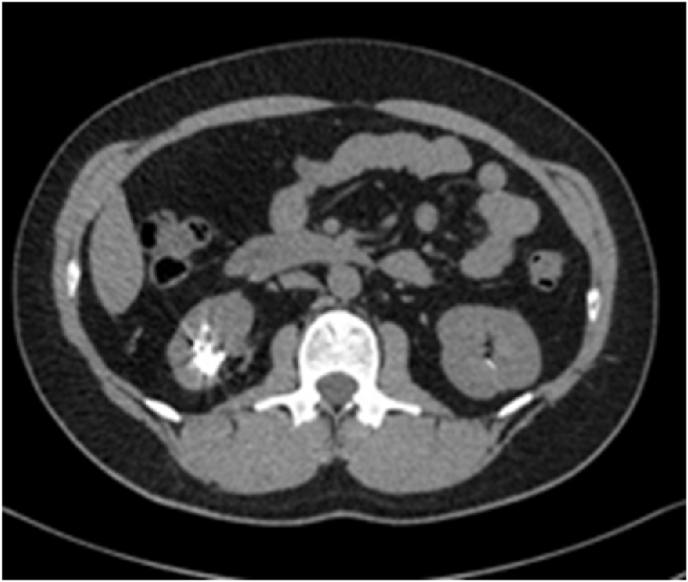
CT abdomen/pelvis (axial view) demonstrated a right renal calculus with an adjacent hyperdense linear density.

Despite initial resolution, the patient later developed recurrent ipsilateral renal stones and underwent ureteroscopy for stone management. Computed tomography of the kidneys, ureters, and bladder (CT KUB) demonstrated a right renal calculus with an adjacent hyperdense linear density suggestive of a foreign body (Fig. 4).

Intraoperatively, a metallic wire-like foreign body protruding into the renal pelvis with stone encrustation along its surface was visualized and identified as a migrated embolization coil. Laser lithotripsy was performed to ablate the exposed portion of the coil and associated stone encrustation. Fragmented portions of the coil were subsequently removed using a stone basket, while the segment of the coil embedded within the renal parenchyma was left in situ. No intraoperative complications or significant bleeding occurred (Fig. 5a, Fig. 5b-ca–c).

**Fig. 5a:**
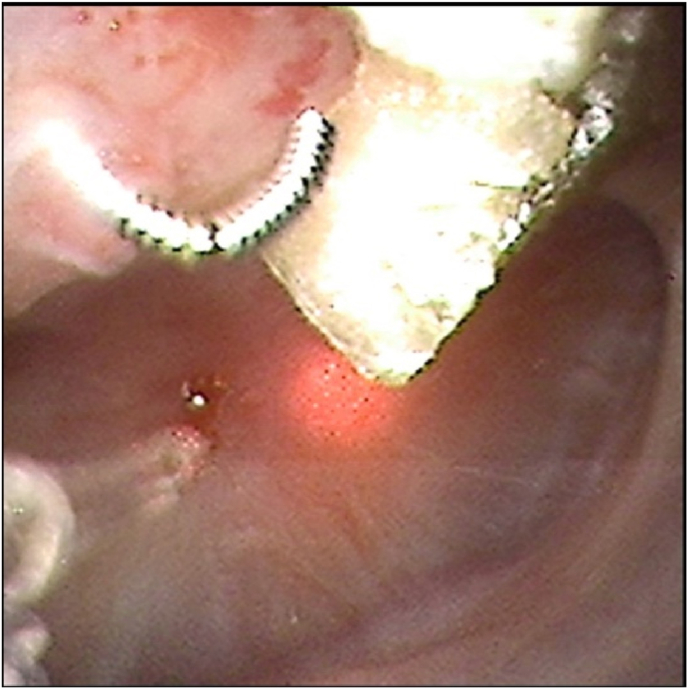
Intraoperative picture showing the calyceal stone with the migrated embolization coil nidus.

**Fig. 5b-c:**
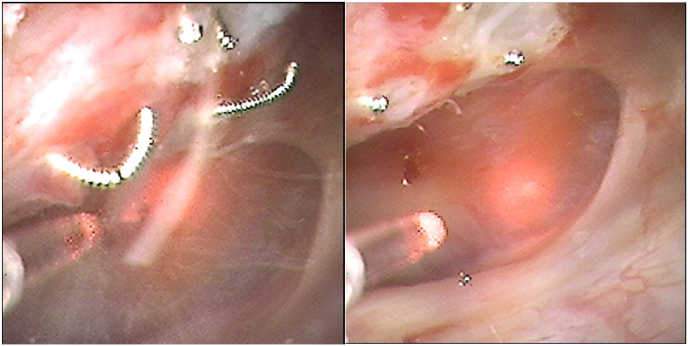
Laser lithotripsy was performed for removal of the stone and the coil.

## 3 Discussion

Migration of embolization coils into the urinary collecting system is a rare but clinically significant event. Proposed mechanisms include gradual erosion through the renal parenchyma or direct communication created by a prior PCNL access tract 1, 2, 3. Once exposed to urine, the coil may act as a nidus for mineral deposition, leading to recurrent calculi.^1^^,^^4^

The material composition and design of embolization coils may further influence stone formation following migration into the collecting system. Modern detachable coils, including the Medtronic Concerto™ 3D coil, incorporate synthetic fibers such as nylon or polylactic-co-glycolic acid to increase surface area and promote thrombogenesis. While these properties are advantageous for vascular occlusion, exposure of fibered coil material to urine may enhance crystal adherence and encrustation.^1^^,^^2^ In the present case, the nylon fiber–containing coil likely facilitated stone formation once intraluminal exposure occurred, particularly in the setting of cystinuria.

It is noteworthy that this patient had an underlying cystine stone disease, which is associated with a high intrinsic risk of recurrence. However, metabolic predisposition alone may not fully explain the pattern of recurrent ipsilateral stone formation observed in this case. Previous reports have demonstrated that migrated embolization coils can serve as a fixed foreign body nidus, resulting in focal and refractory stone formation within the affected renal unit.^1^^,^^4^^,^^5^ In this patient, recurrent stones were consistently found encrusting the embolization coil in the same kidney, supporting the role of coil migration as a contributing mechanical factor rather than metabolic recurrence alone.

Complete removal of an embolization coil may not always be feasible or safe, particularly when the coil is deeply embedded within the renal parenchyma. Aggressive extraction in such circumstances carries a substantial risk of hemorrhage.^3^ Therefore, a tailored endoscopic approach focusing on complete stone clearance and ablation or removal of the exposed intraluminal portion of the coil may represent a reasonable and safe management strategy.^3^^,^^4^ This approach balances the risk of recurrent stone formation against the potential morbidity associated with extensive coil extraction.

Long-term surveillance remains important, as residual embedded coil material may theoretically serve as a future nidus if further migration or exposure occurs.^4^

## 4 Conclusion

Embolization coil migration into the renal collecting system is a rare complication that may cause recurrent stone disease 1, 2, 3, 4. Urologists should maintain a high index of suspicion in patients presenting with recurrent stones following renal arterial embolization. Prompt recognition and appropriate endoscopic management can lead to favorable outcomes, particularly in patients with underlying metabolic stone disorders.

## CRediT authorship contribution statement

**Thanisorn Pattanasuwon:** Writing – original draft, Writing – review & editing. **Maria Camila Vargas:** Writing – review & editing. **Manoj Monga:** Writing – review & editing.

## Financial disclosures

This research did not recieve any specific grant from funding agencies in the public, commerical, or not-for-profit-sectors.

## Declaration of generative AI and AI-assisted technologies in the manuscript preparation process

During the preparation of this work the author(s) used ChatGPT in order to check grammar and assist with sentence phrasing. After using this tool/service, the author(s) reviewed and edited the content as needed and take(s) full responsibility for the content of the published article.

## Conflict of interests

The authors declare that there is no conflict of interest regarding the publication of this paper.
